# Change-points in antibiotic consumption in the community, European Union/European Economic Area, 1997–2017

**DOI:** 10.1093/jac/dkab179

**Published:** 2021-08-01

**Authors:** Robin Bruyndonckx, Ana Hoxha, Chantal Quinten, Girma Minalu Ayele, Samuel Coenen, Ann Versporten, Niels Adriaenssens, Arno Muller, Ole Heuer, Dominique L Monnet, Herman Goossens, Geert Molenberghs, Klaus Weist, Niel Hens, the ESAC-Net study group, Reinhild Strauss, Eline Vandael, Stefana Sabtcheva, Marina Payerl-Pal, Isavella Kyriakidou, Jiří Vlček, Ute Wolff Sönksen, Elviira Linask, Emmi Sarvikivi, Philippe Cavalié, Karin Gröschner, Flora Kontopidou, Mária Matuz, Gudrunw Aspelund, Gudrun Oza, Filomena Fortinguerra, Andis Seilis, Jolanta Kuklytė, Marcel Bruch, Peter Zarb, Stephanie Natsch, Hege Salvesen Blix, Anna Olczak-Pieńkowska, Ana Silva, Ionel Iosif, Tomáš Tesař, Milan Čižman, Mayte Alonso Herreras, Vendela Bergfeldt, Berit Müller-Pebody

**Affiliations:** 1 Laboratory of Medical Microbiology, Vaccine & Infectious Disease Institute (VAXINFECTIO), University of Antwerp, Antwerp, Belgium; 2 Interuniversity Institute for Biostatistics and statistical Bioinformatics (I-BIOSTAT), Data Science Institute, Hasselt University, Hasselt, Belgium; 3 Sciensano, Brussels, Belgium; 4 Disease Programmes Unit, European Centre for Disease Prevention and Control, Stockholm, Sweden; 5 Allergan Plc, 5 Giralda Farms, Madison NJ, USA; 6 Centre for General Practice, Department of Family Medicine & Population Health (FAMPOP), University of Antwerp, Antwerp, Belgium; 7 Interuniversity Institute for Biostatistics and statistical Bioinformatics (I-BIOSTAT), Catholic University of Leuven, Leuven, Belgium; 8 Centre for Health Economic Research and Modelling Infectious Diseases (CHERMID), Vaccine & Infectious Disease Institute, University of Antwerp, Antwerp, Belgium

## Abstract

**Objectives:**

Surveillance of antibiotic consumption in the community is of utmost importance to inform and evaluate control strategies. Data on two decades of antibiotic consumption in the community were collected from 30 EU/European Economic Area (EEA) countries. This article reviews temporal trends and the presence of abrupt changes in subgroups of relevance in antimicrobial stewardship.

**Methods:**

For the period 1997–2017, data on yearly antibiotic consumption in the community, aggregated at the level of the active substance, were collected using the WHO ATC classification and expressed in DDD (ATC/DDD index 2019) per 1000 inhabitants per day. We applied a range of non-linear mixed models to assess the presence of changes in the consumption of antibacterials for systemic use (ATC group J01) and eight antibiotic subgroups.

**Results:**

For the majority of the studied groups, a country-specific change-point model provided the best fit. Depending on the antibiotic group/subgroup and on the country, change-points were spread out between 2000 and 2013.

**Conclusions:**

Due to the heterogeneity in antibiotic consumption in the community across EU/EEA countries, a country-specific change-point model provided the better fit. Given the limitations of this model, our recommendation for the included countries is to carefully interpret the country-specific results presented in this article and to use the tutorial included in this series to conduct their own change-point analysis when evaluating the impact of changes in regulations, public awareness campaigns, and other national interventions to improve antibiotic consumption in the community.

## Introduction

Since their discovery, antibiotics have played an important role in the treatment of bacterial infections. Access to effective antibiotics remains of utmost importance in modern healthcare.^1^ However, overuse and misuse of antibiotics have been identified as important factors leading to the increase of bacterial resistance.^2–5^ Because the therapeutic options for treatment of multidrug-resistant bacterial infections are limited, they represent a major public health threat with prolonged hospital stays, increased health care costs and increased mortality.^6–8^ Therefore, surveillance of antibiotic consumption in both the community (i.e. primary care sector) and hospital setting is crucial in order to inform and evaluate strategies for prevention and control of antibiotic resistance.

In 2001, the European Commission funded the European Surveillance of Antimicrobial Consumption (ESAC) project with the aim of collecting comparable and reliable data on antibiotic consumption in Europe. In 2011, this surveillance activity continued as the European Surveillance of Antimicrobial Consumption Network (ESAC-Net),^9^ managed and hosted by ECDC. Using data collected through these surveillance networks, trends of antibiotic consumption in the community (1997–2009) have been studied for the different antibiotic subgroups.^10–14^ In 2013, Minalu *et al.*^15^ proposed a change-point model using a Bayesian framework to improve model fit by allowing for abrupt changes in tetracycline consumption. The approach suggested by Minalu *et al.*^15^ has previously been used to update two series of articles on antibiotic consumption in the community: 1997–2009^16–21^ and 1997–2003.^10–14^^,^^22–27^ In that update, statistical modelling focused on assessing the occurrence of change-points from the EU/European Economic Area (EEA) perspective, which is reflected by the use of common change-point(s) for the participating countries. However, when focusing on explaining as much of the observed variability as possible, regardless of the perspective, other models, e.g. models including country-specific change-points, might provide a better fit to the data.

In this article, we apply a range of models to data collected through ESAC and ESAC-Net on the consumption of antibacterials for systemic use (ATC J01) and of eight subgroups in the community during 1997–2017.

## Methods

### Data

The methods for collecting and analysing data on antibiotic consumption in the community are described in the introductory article of this series.^28^ In summary, data on antibiotic consumption, aggregated at the level of the active substance, were collected in accordance with the ATC classification defined by the WHO Collaborating Centre for Drug Statistics Methodology.^29^ Data on consumption expressed in DDD (ATC/DDD index 2019)^29^ per 1000 inhabitants per day were available for 30 EU/EEA countries for the period 1997–2017. To limit difficulties in model convergence, countries that reported consumption data for <60% of the total number of years included in the study (i.e. <13 years) were excluded from the change-point analysis.

In this article, we focused on antibacterials for systemic use (ATC J01) and eight specific subgroups: tetracyclines (J01A), β-lactamase-sensitive penicillins (J01CE; narrow-spectrum penicillins) and β-lactamase-resistant penicillins (J01CF; penicillinase-resistant penicillins), cephalosporins (J01DB, J01DC, J01DD and J01DE), combinations of sulphonamides and trimethoprim (J01EE), macrolides (J01FA), fluoroquinolones (J01MA), penicillins with extended spectrum (J01CA; extended-spectrum penicillins) and combinations of penicillins, including β-lactamase inhibitors (J01CR; combinations of penicillins), and nitrofuran derivatives (J01XE). These subgroups were selected because they represent first-line antibiotic treatments or are recommended for use in severe and multidrug-resistant bacterial infections.^30^

### Statistical analysis

In this manuscript, we focused on detecting changes in antibiotic consumption by country, rather than on detecting common changes for the EU/EEA as a whole. Therefore, the statistical analysis deviates slightly from the approach followed in the other articles of this series.^22^^,^^31^ Rather than using quarterly data on antibiotic consumption, we used the more complete yearly data for this manuscript. We considered the following models that are discussed in detail in the tutorial of this series:^31^

*Model 1*: Mixed model without change-points;

*Model 2*: Mixed model with one common change- point (C_1_);

*Model 3*: Mixed model with two common change-points (C_1_ and C_2_ with C_1_<C_2_);

*Model 4*: Mixed model with three common change- points (C_1_, C_2_ and C_3_ with C_1_<C_2_<C_3_).

In addition, we considered the following model:

*Model 5:* Mixed model with one country-specific change- point (c_i_),

where the common change-point (C1) from Model 2 was replaced by a change-point for which the location is country-specific and data-driven (c_i_). To reflect our lack of prior knowledge on the location of this country-specific change-point, we used a uniform distribution over the whole time range.^15^

Model selection was based on the Deviance Information Criterion (DIC),^32^ with trace plots used to verify convergence of the fitted models. Significance was based on 95% Bayesian credible intervals which are intervals from the posterior distribution within which the unobserved parameters fall with 95% probability.^33^ We assumed that the less complete series were for countries that had not joined the network from its start, missed intermittent data calls, or had not yet submitted their data for the more recent years.

## Results

**Figure 1.**
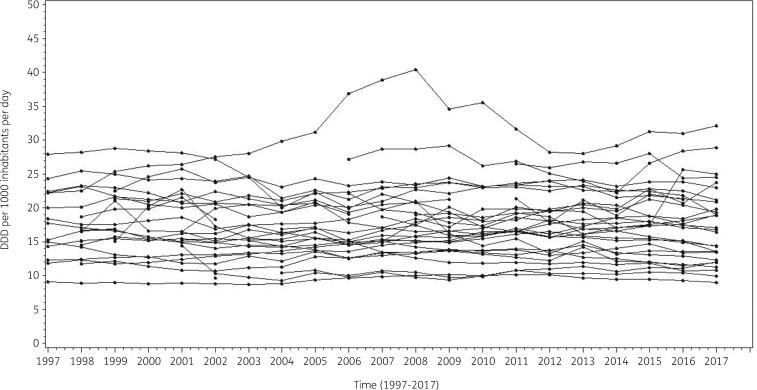
Evolution of consumption of antibacterials for systemic use (ATC J01) in the community, expressed in DDD (ATC/DDD index 2019) per 1000 inhabitants per day for 30 EU/EEA countries, 1997–2017 (unlabelled lines).

The longitudinal profiles for the 30 EU/EEA countries demonstrated that there was heterogeneity both between and within countries (Figure 1). The lowest consumption of antibacterials for systemic use (ATC J01) was recorded in the Netherlands in 2003 (8.72 DDD per 1000 inhabitants per day) while the highest consumption was recorded in Greece in 2008 (40.39 DDD per 1000 inhabitants per day).

Countries reporting antibiotic consumption for <60% of the included years (<13 years), i.e. Romania (8 years reported; community and hospital sector combined), Cyprus (12 years reported; community and hospital sector combined), Malta (11 years reported) and Lithuania (12 years reported), were excluded from further analysis in order to minimize convergence issues.

**Figure 2.**
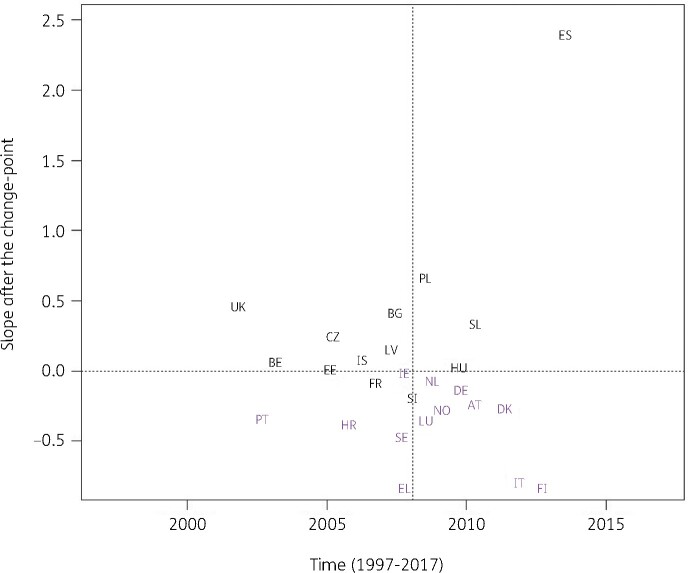
Estimated country-specific trend after the change-point versus location of the country-specific change-point for the consumption of antibacterials for systemic use (ATC J01) in the community obtained from fitting Model 5 on yearly data from 26 EU/EEA countries, 1997–2017. Dotted lines indicate averages. Country labels in black indicate an increase in the trend after versus before the change-point. Country labels in purple indicate a decrease in the trend after versus before the change-point. For Spain, private prescription data are included from 2016 onwards. Abbreviations: AT, Austria; BE, Belgium; BG, Bulgaria; HR, Croatia; CZ, Czechia; DK, Denmark; EE, Estonia; FI, Finland; FR, France; DE, Germany; EL, Greece; HU, Hungary; IS, Iceland; IE, Ireland; IT, Italy; LV, Latvia; LU, Luxembourg; NL, the Netherlands; NO, Norway; PL, Poland; PT, Portugal; SK, Slovakia; SI, Slovenia; ES, Spain; SE, Sweden; UK, United Kingdom.

When modelling consumption data for antibacterials for systemic use (ATC J01), the best model fit was obtained for a model including country-specific change-points (Model 5; Table 1). Consumption of antibacterials for systemic use significantly increased, between 1997 and the country-specific change-point for Denmark, Greece and Italy while it significantly decreased for Bulgaria, France, Hungary and Iceland. After the change-point, a significant increase was observed for Bulgaria, Poland, Spain (private prescriptions included from 2016 onwards) and the United Kingdom while a significant decrease was observed for Croatia, Finland, Greece, Italy and Portugal. Change-points were located between 2002 and 2013, with the majority of the countries’ change-points located around 2008 (Figure 2).

**Table 1.** Deviance Information Criterion for the five models fitted to data on antibiotic consumption of antibacterials for systemic use (ATC J01) and eight selected subgroups of antibiotics

ATC CODE	Model 1 (no CP)	Model 2 (1 CP)	Model 3 (2 CPs)	Model 4 (3 CPs)	Model 5 (country-specific CP)	Location of CPs for the best model
J01	1892.15	1635.64	1519.93	NC	**1518.59**	2002–2013
J01A	145.93	−109.96	**−313.71**	NC	−307.14	2004 and 2011
J01CE & J01CF	324.33	177.77	**7.28**	NC	115.45	2001 and 2006
J01DB to J01DE	257.59	42.79	−41.08	NC	**−97.67**	2001–2013
J01EE	−296.4	−526.29	NC	NC	**−801.08**	2001–2013
J01FA	888.84	236.97	167.86	NC	**−44.6**	2000–2012
J01MA	−102.15	−301.67	−345.56	NC	**−440.87**	2001–2010
J01CA & J01CR	963.83	738.45	650.88	579.56	**425.99**	2005–2013
J01XE	−172.27	−215.33	NC	NC	**−310.22**	2003–2012

Bold font indicates the best fitting model.

CP, change-point; NC, no convergence was obtained; J01, antibacterials for systemic use; J01A, tetracyclines; J01CE, narrow-spectrum penicillins; J01CF, penicillinase-resistant penicillins; J01DB to J01DE, cephalosporins; J01EE, combinations of sulphonamides and trimethoprim; J01FA, macrolides; J01MA, fluoroquinolones; J01CA, extended-spectrum penicillins; J01CR, combinations of penicillins; J01XE, nitrofuran derivatives.

For tetracyclines (J01A), the best model fit was obtained for the model containing two common change-points: one in 2004 and one in 2011 (Model 3; Table 1). In 1997, tetracycline consumption was estimated at 2.861 (SE 0.265) DDD per 1000 inhabitants per day. In general, tetracycline consumption decreased over time (−0.054, SE 0.019, DDD per 1000 inhabitants per day per year) up to 2004, after which it did not change significantly [+0.030, SE 0.030, DDD per 1000 inhabitants per day per year up to 2011, then −0.013 (SE 0.029) DDD per 1000 inhabitants per day per year afterwards]. Significant increases were observed between 1997 and 2004 for France, Germany and Ireland while significant decreases were observed for Belgium, Bulgaria, Estonia, Finland, Hungary, Luxembourg, Norway, Poland, Portugal and Slovenia. Between 2005 and 2011, significant increases were observed for Denmark, Finland, the Netherlands, Norway and the United Kingdom while significant decreases were observed for Bulgaria, Croatia, Czechia, Estonia, Germany, Greece, Hungary, Ireland, Poland, Slovakia and Slovenia. After 2011, significant increases were observed for Greece, Slovakia, Spain (private prescriptions included from 2016 onwards) and the United Kingdom while significant decreases were observed for Austria, Croatia, Denmark, Estonia, Finland, Germany, Luxembourg, the Netherlands and Sweden.

For narrow-spectrum and penicillinase-resistant penicillins (J01CE and J01CF), the best model fit was obtained for the model containing two common change-points: one in 2001 and one in 2006 (Model 3; Table 1). In 1997, consumption of narrow-spectrum and penicillinase-resistant penicillins was estimated at 1.980 (SE 0.347) DDD per 1000 inhabitants per day. In general, consumption did not change significantly over time [−0.018, SE 0.040, DDD per 1000 inhabitants per day per year up to 2001, then −0.077 (SE 0.071) DDD per 1000 inhabitants per day per year up to 2006, and −0.045 (SE 0.084) DDD per 1000 inhabitants per day per year afterwards]. Significant increases were observed between 1997 and 2001 for Denmark and Slovakia while significant decreases were observed for Finland, Iceland and Poland. Between 2002 and 2006, significant increases were observed for Denmark and Greece while significant decreases were observed for Bulgaria, Finland, Germany, Iceland, Poland, Slovakia and Slovenia. After 2006, significant increases were observed for Ireland and the United Kingdom while significant decreases were observed for Croatia, Denmark, Greece, Hungary, Iceland, Norway, Slovakia, Slovenia and Sweden.

**Figure 3.**
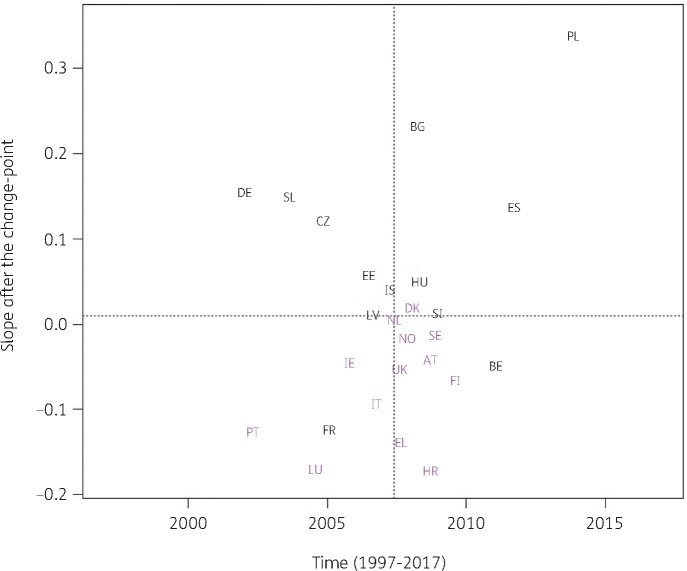
Estimated country-specific trend after the change-point versus location of the country-specific change-point for the consumption of cephalosporins (ATC J01DB, J01DC, J01DD and J01DE) in the community obtained from fitting Model 5 on yearly data from 26 EU/EEA countries, 1997–2017. Dotted lines indicate averages. Country labels in black indicate an increase in the trend after versus before the change-point. Country labels in purple indicate a decrease in the trend after versus before the change-point. For Spain, private prescription data are included from 2016 onwards. AT, Austria; BE, Belgium; BG, Bulgaria; HR, Croatia; CZ, Czechia; DK, Denmark; EE, Estonia; FI, Finland; FR, France; DE, Germany; EL, Greece; HU, Hungary; IS, Iceland; IE, Ireland; IT, Italy; LV, Latvia; LU, Luxembourg; NL, the Netherlands; NO, Norway; PL, Poland; PT, Portugal; SK, Slovakia; SI, Slovenia; ES, Spain; SE, Sweden; UK, United Kingdom.

For the other subgroups, the best model fit was obtained for a country-specific change-point model (Model 5, Table 1). Consumption of cephalosporins (J01DB, J01DC, J01DD and J01DE) significantly increased between 1997 and the country-specific change-point for Greece and Poland while it significantly decreased for Belgium, France, Hungary and Spain (private prescriptions included from 2016 onwards). After the change-point, a significant increase was observed for Bulgaria, Czechia, Germany, Poland, Slovakia and Spain (private prescriptions included from 2016 onwards) while a significant decrease was observed for Croatia, France, Greece and Luxembourg. Change-points were located between 2001 and 2013, with the majority of the countries’ change-points located around 2008 (Figure 3).

**Figure 4.**
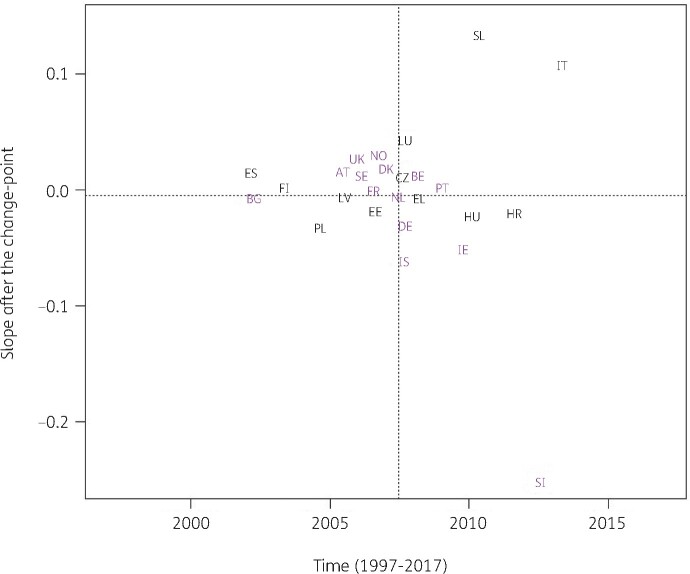
Estimated country-specific trend after the change-point versus location of the country-specific change-point for the consumption of combinations of sulphonamides and trimethoprim (ATC J01EE) in the community obtained from fitting Model 5 on yearly data from 26 EU/EEA countries, 1997–2017. Dotted lines indicate averages. Country labels in black indicate an increase in the trend after versus before the change-point. Country labels in purple indicate a decrease in the trend after versus before the change-point. For Spain, private prescription data are included from 2016 onwards. AT, Austria; BE, Belgium; BG, Bulgaria; HR, Croatia; CZ, Czechia; DK, Denmark; EE, Estonia; FI, Finland; FR, France; DE, Germany; EL, Greece; HU, Hungary; IS, Iceland; IE, Ireland; IT, Italy; LV, Latvia; LU, Luxembourg; NL, the Netherlands; NO, Norway; PL, Poland; PT, Portugal; SK, Slovakia; SI, Slovenia; ES, Spain; SE, Sweden; UK, United Kingdom.

Consumption of combinations of sulphonamides and trimethoprim (J01EE) significantly increased between 1997 and the country-specific change-point for Bulgaria while it significantly decreased for Austria, Belgium, Czechia, Estonia, Finland, Greece, Hungary, Iceland, Italy, Luxembourg, Poland, Portugal, Slovakia, Slovenia and Spain (private prescriptions included from 2016 onwards). After the change-point, a significant increase was observed for Slovakia while a significant decrease was observed for Bulgaria, Poland and Slovenia. Change-points were located between 2001 and 2013, with the majority of the countries’ change-points located around 2007 (Figure 4).

**Figure 5.**
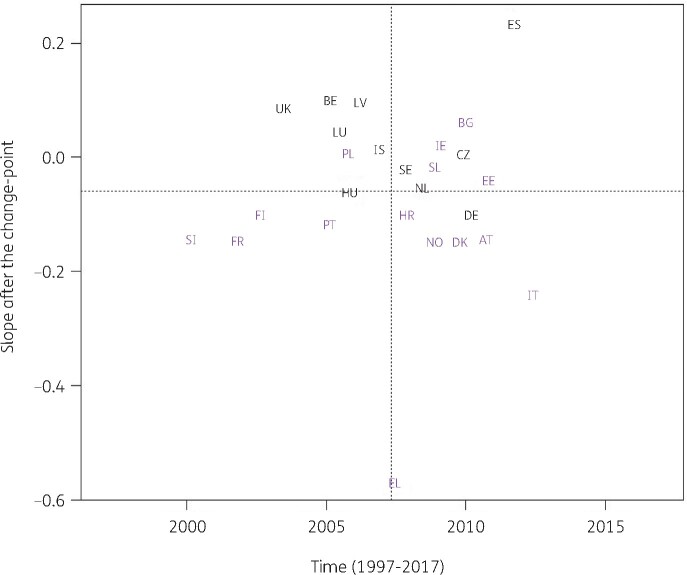
Estimated country-specific trend after the change-point versus location of the country-specific change-point for the consumption of macrolides (ATC J01FA) in the community obtained from fitting Model 5 on yearly data from 26 EU/EEA countries, 1997–2017. Dotted lines indicate averages. Country labels in black indicate an increase in the trend after versus before the change-point. Country labels in purple indicate a decrease in the trend after versus before the change-point. For Spain, private prescription data are included from 2016 onwards. AT, Austria; BE, Belgium; BG, Bulgaria; HR, Croatia; CZ, Czechia; DK, Denmark; EE, Estonia; FI, Finland; FR, France; DE, Germany; EL, Greece; HU, Hungary; IS, Iceland; IE, Ireland; IT, Italy; LV, Latvia; LU, Luxembourg; NL, the Netherlands; NO, Norway; PL, Poland; PT, Portugal; SK, Slovakia; SI, Slovenia; ES, Spain; SE, Sweden; UK, United Kingdom.

Consumption of macrolides (J01FA) significantly increased between 1997 and the country-specific change-point for Bulgaria, Croatia, Estonia, Greece, Ireland, Poland, Portugal and Slovakia while it significantly decreased for Belgium, Luxembourg and Spain (private prescriptions included from 2016 onwards). After the change-point, a significant increase was observed for Belgium, Luxembourg, Spain (private prescriptions included from 2016 onwards) and the United Kingdom while a significant decrease was observed for Austria, Croatia, Denmark, Finland, Greece, Italy, Norway and Portugal. Change-points were located between 2000 and 2012, with the majority of the countries’ change-points located around 2008 (Figure 5).

**Figure 6.**
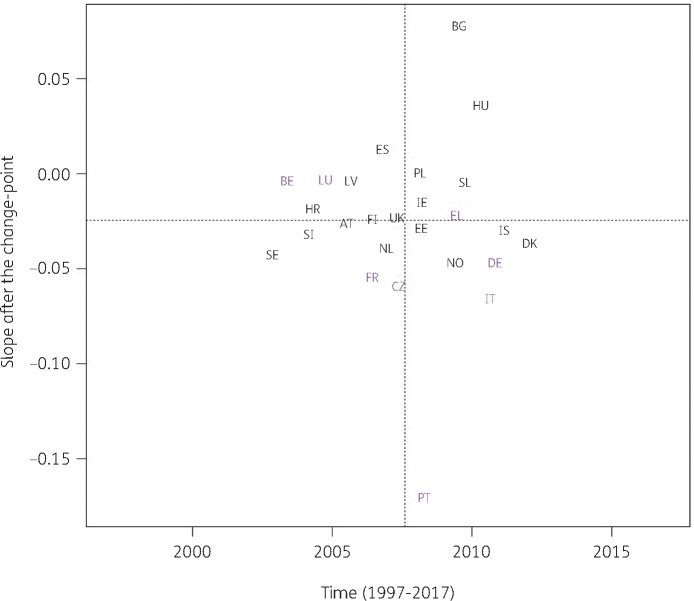
Estimated country-specific trend after the change-point versus location of the country-specific change-point for the consumption of fluoroquinolones (ATC J01MA) in the community obtained from fitting Model 5 on yearly data from 26 EU/EEA countries, 1997–2017. Dotted lines indicate averages. Country labels in black indicate an increase in the trend after versus before the change-point. Country labels in purple indicate a decrease in the trend after versus before the change-point. For Spain, private prescription data are included from 2016 onwards. AT, Austria; BE, Belgium; BG, Bulgaria; HR, Croatia; CZ, Czechia; DK, Denmark; EE, Estonia; FI, Finland; FR, France; DE, Germany; EL, Greece; HU, Hungary; IS, Iceland; IE, Ireland; IT, Italy; LV, Latvia; LU, Luxembourg; NL, the Netherlands; NO, Norway; PL, Poland; PT, Portugal; SK, Slovakia; SI, Slovenia; ES, Spain; SE, Sweden; UK, United Kingdom.

Consumption of fluoroquinolones (J01MA) significantly increased between 1997 and the country-specific change-point for all participating countries except Croatia, Latvia, the Netherlands, Portugal, Slovenia, Sweden and the United Kingdom. After the change-point, a significant decrease was observed for Czechia, France, Germany, Italy, Portugal and Sweden. Change-points were located between 2001 and 2010, with the majority of the countries’ change-points located around 2006 (Figure 6).

**Figure 7.**
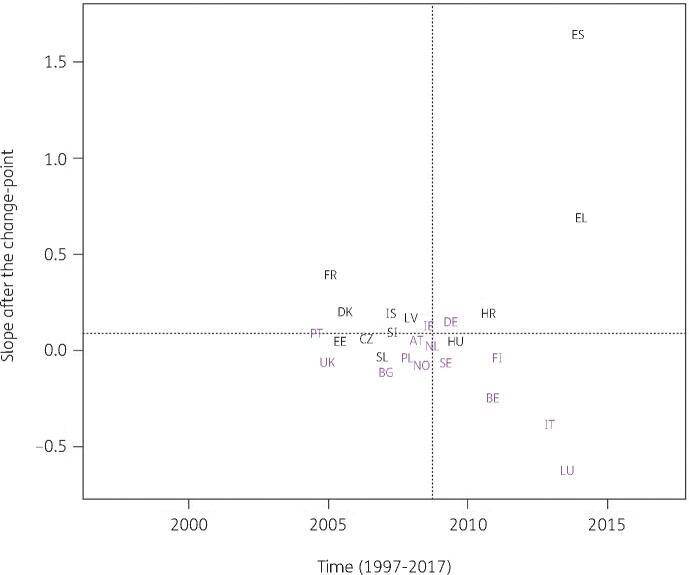
Estimated country-specific trend after the change-point versus location of the country-specific change-point for the consumption of extended-spectrum penicillins and of combinations of penicillins combined (ATC J01CA and J01CR) in the community obtained from fitting Model 5 on yearly data from 26 EU/EEA countries, 1997–2017. Dotted lines indicate averages. Country labels in black indicate an increase in the trend after versus before the change-point. Country labels in purple indicate a decrease in the trend after versus before the change-point. For Spain, private prescription data are included from 2016 onwards. AT, Austria; BE, Belgium; BG, Bulgaria; HR, Croatia; CZ, Czechia; DK, Denmark; EE, Estonia; FI, Finland; FR, France; DE, Germany; EL, Greece; HU, Hungary; IS, Iceland; IE, Ireland; IT, Italy; LV, Latvia; LU, Luxembourg; NL, the Netherlands; NO, Norway; PL, Poland; PT, Portugal; SK, Slovakia; SI, Slovenia; ES, Spain; SE, Sweden; UK, United Kingdom.

Consumption of extended-spectrum penicillins and combinations of penicillins (J01CA and J01CR) significantly increased between 1997 and the country-specific change-point for Belgium, Greece, Italy, Luxembourg, Portugal and Spain (private prescriptions included from 2016 onwards) while it significantly decreased for Estonia, France, Hungary, Iceland and Slovakia. After the change-point, a significant increase was observed for Croatia, France, Greece, Iceland and Spain (private prescriptions included from 2016 onwards) while a significant decrease was observed for Belgium, Italy and Luxembourg. Change-points were located between 2005 and 2013, with the majority of the countries’ change-points located around 2008 (Figure 7).

**Figure 8.**
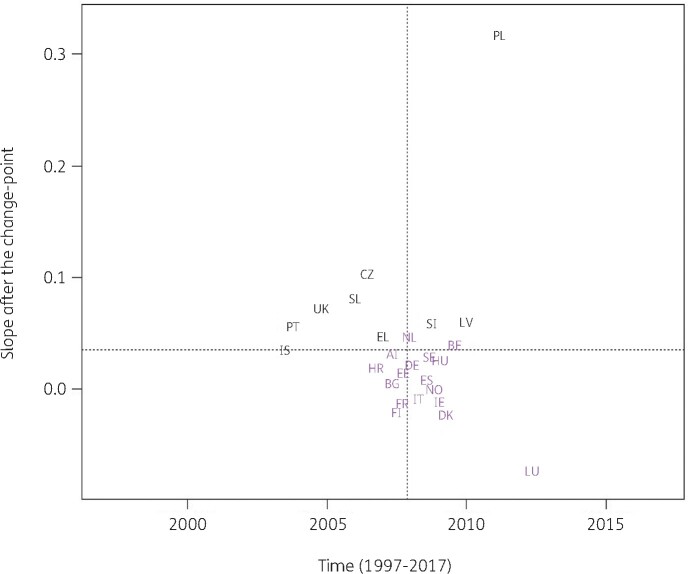
Estimated country-specific trend after the change-point versus location of the country-specific change-point for the consumption of nitrofuran derivatives (ATC J01XE) in the community obtained from fitting Model 5 on yearly data from 26 EU/EEA countries, 1997–2017. Dotted lines indicate averages. Country labels in black indicate an increase in the trend after versus before the change-point. Country labels in purple indicate a decrease in the trend after versus before the change-point. For Spain, private prescription data are included from 2016 onwards. For Ireland, consumption of nitrofurantoin (J01XE01) was not included. AT, Austria; BE, Belgium; BG, Bulgaria; HR, Croatia; CZ, Czechia; DK, Denmark; EE, Estonia; FI, Finland; FR, France; DE, Germany; EL, Greece; HU, Hungary; IS, Iceland; IE, Ireland; IT, Italy; LV, Latvia; LU, Luxembourg; NL, the Netherlands; NO, Norway; PL, Poland; PT, Portugal; SK, Slovakia; SI, Slovenia; ES, Spain; SE, Sweden; UK, United Kingdom.

Consumption of nitrofuran derivatives (J01XE) significantly increased between 1997 and the country-specific change-point for Luxembourg and Poland while it significantly decreased for Iceland. After the change-point, a significant increase was observed for Czechia, Iceland, Poland and Slovenia. Change-points were located between 2003 and 2012, with the majority of the countries’ change-points located around 2008 (Figure 8).

Including a third change-point (Model 4) resulted in non-convergence for all subgroups under study, indicating that the maximum number of common change-points was two.^15^

## Discussion

For the majority of the subgroups under study, the country-specific change-point model (Model 5) provided the best fit. This is not surprising given the heterogeneity in antibiotic consumption in the community between EU/EEA countries. Previous studies on antibiotic consumption in Europe have pointed out this heterogeneity between EU/EEA countries with marked North-to-South and West-to-East gradients for both antibiotic consumption and resistance.^34^

Data on tetracycline consumption in the community show that there are two common change-points: one in 2004 and one in 2011. However, the evolution of tetracycline consumption over time is country-specific with some countries showing increasing, and other decreasing, tetracycline consumption in the periods 1997–2004, 2005–2011 and 2012–2017. Similarly for narrow-spectrum and penicillinase-resistant penicillins, two common change-points were observed: one in 2001 and one in 2006. However, in addition for this subgroup, the evolution over time was country-specific.

For antibacterials for systemic use and for the remaining subgroups, models with one country-specific change-point provided a better fit. These country-specific change-points were spread out between 2001 and 2013. For most countries, the trend in antibiotic consumption after the change-point remained quite close to the general trend. However, exceptions were the steep decrease in macrolide consumption in Greece after 2007 (Figure 5) and the steep increase in consumption of extended-spectrum penicillins and combinations of penicillins in Spain (private prescriptions included from 2016 onwards) after 2013 (Figure 7). For macrolide consumption, both increases and decreases in consumption were observed with the largest increase reported for Spain (private prescriptions included from 2016 onwards). Similarly for extended-spectrum penicillins and combinations of penicillins, both increases and decreases in consumption were observed with the largest decrease reported for Luxembourg.

Consumption of antibacterials for systemic use increased in some countries, while it decreased in other countries, with the largest increase reported for Spain (private prescriptions included from 2016 onwards) and the largest decrease reported for Finland (Figure 2). For cephalosporins, consumption increased for some countries, while it decreased for other countries. The largest increase was reported for Poland and the largest decrease for Luxembourg (Figure 3). A limiting factor of the analysis of this subgroup is that cephalosporin consumption data were pooled but consumption of the different generations of cephalosporins may have varied over time.^24^^,^^35^

For combinations of sulphonamides and trimethoprim (Figure 4) and fluoroquinolones (Figure 6), both increases and decreases were observed for individual countries. The largest increases were reported for Slovakia and Bulgaria, respectively, while the largest decreases were reported for Slovenia and Portugal, respectively. Fluoroquinolones are commonly used antibiotics, which are often prescribed for non-approved conditions or inappropriately prescribed for viral infections.^26^ Additionally, their use should be restricted due to potential severe side-effects.^36^ The increasing trend of fluoroquinolone consumption, particularly in Bulgaria and Hungary suggests increasing inappropriate use of this important group of antibiotics and is worrisome.^34^^,^^37^

For the nitrofuran derivatives (Figure 8), no significant change was observed for most countries while a significant increase was reported for Czechia, Iceland, Poland and Slovenia. This could be related to more appropriate prescribing for urinary tract infections as recommended by many guidelines or an increased interest in nitrofuran derivatives to compensate for antibiotic resistance to other commonly prescribed antibiotics such as fluoroquinolones and combinations of sulphonamides and trimethoprim.^38^^,^^39^ However, the increase in consumption of nitrofuran derivatives was only accompanied by a significant decrease in combinations of sulphonamides and trimethoprim in Poland and Slovenia, but not in Czechia or Iceland, and by a significant decrease in consumption of fluoroquinolones for Czechia, but not for Slovenia, Poland or Iceland. Another possible explanation for the increase in nitrofuran derivatives could be over-the-counter availability of nitrofurantoin in several countries.^40^

In order to explain why change-points occurred at specific points in time, three elements should be considered. The first element is the infection rate in consecutive years, with a low (or high) infection rate resulting in low (or high) antibiotic consumption. For example, when analysing yearly consumption data, one calendar year may include zero, one or two seasonal influenza epidemics, which would affect antibiotic consumption. This could be avoided by using quarterly data. Within the infection rate, we need to consider both the bacterial and the viral infection rate as antibiotics are often inappropriately prescribed for viral infections, mainly respiratory tract infections during the winter months. This element could be of particular interest to explain some of the change-points observed for antibiotics that are often overused during, for example, seasonal influenza epidemics.

A second element to be considered when explaining the location of the change-points are actions such as public awareness campaigns on prudent antibiotic use and antibiotic resistance, shortages and imposed restrictions. These could occur at different points in time for individual EU/EEA countries with the aim to reduce unnecessary antibiotic use and therewith the occurrence of resistance.^41^ National evaluations of such campaigns have been reported for Belgium and France.^42–45^ However, we recommend that each country conducts an extensive review on its public awareness campaigns to better understand the reported country-specific change-points.

A third element to take into consideration when interpreting the results of the change-point analysis is the change of package size driven by companies’ packaging practices. In general, over the years, pharmaceutical companies have increased the number of doses per package, which, for some countries, may have had an effect on consumption rates when expressed in DDD per 1000 inhabitants per day.^46–48^

Because the change-point analysis detects the most significant change in trend, care should be taken in comparing detected change-points with previous research. Changing the number of included countries could have an effect on the location of common change-points, and changing the time-frame could have an effect on the locations of both country-specific and common change-points. Our recommendation for the included countries is to interpret their results as presented in this article with caution and use the tutorial included in this series to conduct a change-point analysis when evaluating changes in guidelines or regulations, public awareness campaigns, or other national interventions.^31^

In conclusion, the heterogeneity of antibiotic consumption across EU/EEA countries required country-specific change-points to assess changes in consumption over the period 1997–2017. However, given the above-mentioned limitations of a model containing country-specific change-points, our recommendation is that each individual country conducts its own analysis, based on the tutorial in this series, with its own data and additional contextual information, e.g. influenza epidemics, awareness campaigns and change in package size, when evaluating national interventions to improve antibiotic consumption in the community.
